# A novel GntR-ABC efflux system mediates oxidative stress response, drug resistance, motility and virulence in *Acinetobacter baumannii* ATCC 17978

**DOI:** 10.3389/fmicb.2026.1748186

**Published:** 2026-04-09

**Authors:** Jordi Corral, María Pérez-Varela, Miquel Sánchez-Osuna, Marc Gaona, Susana Campoy, Jordi Barbé, Jesús Aranda

**Affiliations:** 1 Departament de Genètica i de Microbiologia, Facultat de Biociènces, Universitat Autònoma de Barcelona (UAB), Barcelona, Spain; 2 Laboratori de Recerca en Microbiologia i Malalties Infeccioses, Hospital Universitari Parc Taulí, Institut d’Investigació i Innovació Parc Taulí (I3PT−CERCA), Universitat Autònoma de Barcelona, Sabadell, Spain; 3 Institut de Biotecnologia i Biomedicina, Universitat Autònoma de Barcelona, Barcelona, Spain

**Keywords:** ABC efflux pump, *Acinetobacter baumannii*, GntR transcriptional regulator, oxidative stress, pathogenesis

## Abstract

**Introduction:**

*Acinetobacter baumannii* is a highly adaptable opportunistic pathogen, notorious for its persistence in healthcare environments and its remarkable ability to withstand oxidative stress, antimicrobial treatments, and immune defenses.

**Methods:**

In this study, we characterized the role of the GntR family transcriptional regulator AUO97_RS16340 and its associated ABC efflux pump (AUO97_RS16355–RS16360–RS16365), previously identified as part of the OxyR regulon.

**Results:**

Through gain- and loss-of-function analyses, we demonstrate that this GntR regulator positively regulates the expression of the adjacent ABC efflux pump and plays a critical role in tolerance to H_2_O_2_-induced oxidative damage. Furthermore, we show that this efflux system contributes to multiple virulence-associated traits, including antimicrobial resistance, surface-associated motility, and virulence.

**Discussion:**

Our findings reveal a new efflux system that links oxidative stress adaptation, efflux-mediated antimicrobial susceptibility, and pathogenicity in *A. baumannii* strain ATCC 17978. Given the high conservation of the GntR transcriptional regulator and its associated efflux pump across *A. baumannii* genomes, these components represent promising therapeutic targets. Disrupting their activity could sensitize bacteria to oxidative stress and antimicrobials while attenuating virulence, providing a potential strategy to combat multidrug-resistant *A. baumannii*.

## 1 Introduction

*Acinetobacter baumannii* is an opportunistic Gram-negative bacterium frequently associated with a broad spectrum of hospital-acquired infections, including ventilator-associated pneumonia and bloodstream infections in severely ill patients (Lee et al., 2017). Its pathogenic success is largely attributed to its ability to adapt to hostile environments, withstand antimicrobial treatments, and exploit multiple virulence factors that promote survival and adaptability (Harding et al., 2018; Ibrahim et al., 2021). A major global concern associated with *A. baumannii* is the rapid emergence of antimicrobial resistance (AMR), which has become one of the most critical global health threats of the 21st century (Boucher et al., 2009; De Oliveira et al., 2020; Huemer et al., 2020). AMR severely compromises therapeutic efficacy, prolongs disease duration, increases mortality rates, and imposes substantial economic and clinical burdens on healthcare systems. Among nosocomial pathogens, the World Health Organization’s 2024 Bacterial Priority Pathogens List designates carbapenem-resistant *A. baumannii* as a critical priority pathogen, underscoring the urgent need to better understand its mechanisms of resistance and pathogenicity (Miller and Arias, 2024; Sati et al., 2025).

The underlying mechanisms of AMR are diverse and can be broadly classified into: (i) drug inactivation or modification, (ii) target alteration, and (iii) changes in cell permeability (Santajit and Indrawattana, 2016; Yazgan et al., 2018). Among these, efflux pumps play a particularly prominent role by actively extruding antibiotics, thereby reducing their intracellular concentrations and diminishing therapeutic effectiveness (Verma et al., 2021; Lorusso et al., 2022). Beyond resistance, these transporters also mediate the extrusion of toxic compounds, metabolites and signaling molecules and are associated with various virulence-related processes such as motility, quorum sensing, and biofilm formation (Pérez et al., 2012; Ma et al., 2016; Knight et al., 2018; Lorusso et al., 2022).

During infection and under environmental challenges, *A. baumannii* is constantly exposed to reactive oxygen species (ROS), which inflict oxidative damage on essential macromolecules including DNA, lipids, and proteins (Imlay, 2003). These highly reactive molecules are produced by the host immune response to eliminate invading pathogens (Winterbourn et al., 2016; Sato et al., 2019), by antimicrobial agents (Ma et al., 2016; Van Acker and Coenye, 2017), and by hospital disinfectants such as hydrogen peroxide (H_2_O_2_) (Biswas et al., 2018). In addition, ROS are also produced through various environmental and microbial processes (Cabiscol et al., 2000; França et al., 2007). Interestingly, certain bactericidal antibiotics, such as quinolones, aminoglycosides, and *β*-lactams, also promote ROS production, which acts synergistically with their primary mechanisms of action to enhance antibiotic-mediated killing (Dwyer et al., 2014; Brand et al., 2025). For instance, aminoglycosides induce protein misfolding and activate envelope stress responses, leading to the generation of hydroxyl radicals and triggering a conserved oxidative damage-driven death pathway across different antibiotic classes (Dwyer et al., 2009; Lang et al., 2023).

To counteract ROS, bacteria like *A. baumannii* rely on a coordinated oxidative stress response, employing a variety of detoxification enzymes, including superoxide dismutases, catalases, and peroxidases (Cabiscol et al., 2000; Sun et al., 2016; Brand et al., 2025). This response is primarily regulated by the transcription factor OxyR (Hishinuma et al., 2006; Juttukonda et al., 2018). Upon oxidative activation, OxyR undergoes conformational changes that enable it to bind specific promoter regions and induce the transcription of genes involved in ROS detoxification (Sun et al., 2016). In addition to its central role in oxidative stress defense, OxyR has been implicated in the virulence of several plant and animal pathogenic bacteria, including *Xanthomonas campestris* (Charoenlap et al., 2005), *Ralstonia solanacearum* (Flores-Cruz and Allen, 2011), *Corynebacterium diphtheriae* (Cappelli et al., 2022), *Escherichia coli* (Johnson et al., 2013) and *Pseudomonas aeruginosa* (Bae and Cho, 2012; Oh et al., 2021), underscoring its multifaceted role in stress response and pathogenicity.

Beyond the canonical antioxidant enzymes regulated by OxyR (Charoenlap et al., 2005; Hishinuma et al., 2006; Jittawuttipoka et al., 2009; Sun et al., 2016), increasing evidence indicates the involvement of additional, often uncharacterized, genes in the oxidative stress response that fulfill diverse cellular functions (Yoon et al., 2002; Seib et al., 2007; Scribano et al., 2025). Transcriptomic analyses have shown that exposure to H_2_O_2_ triggers extensive transcriptional reprogramming in *A. baumannii* ATCC 17978, affecting the expression of hundreds of genes (Juttukonda et al., 2018). These include not only those encoding ROS-detoxifying enzymes, but also transcriptional regulators, transport systems, and genes associated with other functional categories. Among these, the regulatory gene *AUO97_RS16340* encoding a GntR family transcriptional regulator is strongly upregulated under oxidative stress and located adjacent to a non-characterized ATP-binding cassette (ABC) efflux pump (Coyne et al., 2011; Juttukonda et al., 2018).

This study aims to elucidate the role of this GntR-ABC efflux system, in *A. baumannii* ATCC 17978 strain, in multiple pathogenic-associated phenotypes, including antimicrobial susceptibility, surface-associated motility, intracellular survival within macrophages, and virulence in the *Galleria mellonella* infection model. Collectively, our findings would provide new insights into efflux systems that might integrate oxidative stress adaptation to efflux-mediated functions, highlighting potential therapeutic targets against multidrug-resistant *A. baumannii*.

## 2 Materials and methods

### 2.1 Strains, plasmids and growth conditions

All bacterial strains and plasmids used in this study are listed in Table 1. *E. coli* (DH5α and BL21 (DE3) pLysS), *A. baumannii* ATCC 17978 wild-type (WT), and its derivative strains were routinely cultured at 37 °C in Luria-Bertani (LB) medium with shaking at 180 rpm. When required, antibiotics were added to the medium at the following final concentrations: kanamycin, 50 mg/L; gentamicin, 20 mg/L for *E. coli* and 40 mg/L for *A. baumannii*; chloramphenicol, 34 mg/L; and ampicillin, 50 mg/L. Growth of *A. baumannii* was monitored by inoculating LB broth with an overnight culture at a 1:100 dilution, followed by incubation at 37 °C with shaking at 180 rpm. The optical density at 600 nm (OD_600_) was measured every hour.

**Table 1:** Bacterial strains, eukaryotic cells and plasmids used in this work.

Strain or plasmid	Relevant characteristics	Source or reference
Bacterial strains		
DH5α	*E. coli supE4 ΔlacU169 (ɸ80 ΔlacZ ΔM15) hsdR17, recA1, endA1, gyrA96, thi-1, relA1*	Clontech
BL21 (DE3) pLysS	*E. coli F^−^ dcm ompT lon hsdS(r_B_^−^m_B_^−^) galλ(DE3)* carring pLysS plasmid, Cm^r^	Stratagene
ATCC 17978 WT	*A. baumannii* Wild-type strain	ATCC
GntR (*RS16340*)	ATCC 17978 derivative strain with *AUO97_RS16340::pCR-BluntII-TOPO* disruption, Km^r^, Zeo^r^	This study
GntR (*RS16340*) complemented	*AUO97_RS16340* strain carrying the pBAV1Gm-T5-gfp plasmid with the corresponding gene cloned, Km^r^, Zeo^r^, Gm^r^	This study
ABC permease (*RS16360*)	ATCC 17978 derivative strain with *AUO97_RS16360::pCR-BluntII-TOPO* disruption, Km^r^, Zeo^r^	This study
ABC permease (*RS16360*) complemented	*AUO97_RS16360* strain carrying the pBAV1Gm-T5-gfp plasmid with the corresponding gene cloned, Km^r^, Zeo^r^, Gm^r^	This study
Eukaryotic strains		
J774A.1 TIB-67™	Cell line isolated in 1968 from the ascites of an adult, female mouse with reticulum cell sarcoma	ATCC
Plasmids		
pCR-BluntII-TOPO	Cloning vector, Km^r^, Zeo^r^	Invitrogen
pBAV1Gm-T5-gfp	pBAV1K-T5-*gfp* derivative complementation vector carrying gentamicin cassette, Gm^r^	Corral et al. (2021)
pUA1108	pGEX 4 T-1 derivative plasmid carrying without the GST fusion tag, carrying only the Ptac IPTG-inducible promoter and the lacIq gene; Amp^r^	Mayola et al. (2014)
pGEM-T	Cloning vector, Amp^r^	Promega

Km^r^, Zeo^r^, Gm^r^, Cm^r^ and Amp^r^, stand for resistance to kanamycin, zeocin, gentamicin, chloramphenicol and ampicillin, respectively.

### 2.2 Accession numbers and homologs detection

The gene sequences of *A. baumannii* strain ATCC 17978 (NZ_CP018664) used in this study were retrieved from National Center for Biotechnology Information [NCBI (RRID:SCR_006472)] database and are listed in Table 2. Homologs of AUO97_RS16340 (GntR), AUO97_RS16355, AUO97_RS16360 and AUO97_RS16365 (ABC efflux system) were identified through reciprocal BLASTP (RRID:SCR_001010) using the *A. baumannii* ATCC 17978 (GCF_001593425) proteins as the query. Putative homologs were defined as bidirectional BLASTP (Altschul et al., 1997) hits satisfying stringent criteria for e-value (<1e^−20^) and minimum query coverage (≥75%) in all complete *Acinetobacter* genome assemblies (*n* = 1,479) and reference complete Gammaproteobacteria assemblies (*n* = 1,228) available on the NCBI database as of February 2026, excluding atypical genomes, MAGs and multiscale projects.

**Table 2:** Correspondence between the updated gene annotation (NZ_CP018664) and the previous annotation (CP000521.1) for the genes analyzed in this study from *A. baumannii* strain ATCC 17978.

Protein ID accession number	Gene ID new annotation	Gene ID old annotation	Putative function
WP_001186397.1	*AUO97_RS16335*	*A1S_1716*	Chromate efflux transporter
WP_000080816.1	*AUO97_RS16340*	*A1S_1717*	GntR family transcriptional regulator
WP_000428990.1	*AUO97_RS16345*	*A1S_1718*	Fumarate reductase/succinate dehydrogenase
WP_000864076.1	*AUO97_RS16350*	*A1S_1719*	4Fe-4S dicluster domain-containing protein
WP_001249623.1	*AUO97_RS16355*	*A1S_1720*	ABC transporter substrate-binding protein
WP_000096316.1	*AUO97_RS16360*	*A1S_1721*	ABC transporter permease
WP_000028994.1	*AUO97_RS16365*	*A1S_1722*	ABC transporter ATP-binding protein

### 2.3 Sequence and genomic contexts analysis

Functional annotation was conducted using HMMER (Eddy, 2011), including assignment to Clusters of Orthologous Groups (COG) database (Galperin et al., 2025) and detection of conserved domains through searches against the Pfam database (Paysan-Lafosse et al., 2025). All-against-all pairwise global alignments were performed using the Needleman-Wunsch algorithm (Needleman and Wunsch, 1970).

Genetic arrangements of detected homologs were retrieved from GenBank-formatted genome assemblies, extracting 5,000 bp flanking regions on each side of the AUO97_RS16360 homologs. The corresponding gene products were annotated using HMMER (Eddy, 2011) against the COG database (Galperin et al., 2025). Synteny conservation was assessed using a custom Python script. For each genome, ordered COG annotations of genes within the selected genomic environment were extracted and compared using the *A. baumannii* ATCC 17978 (GCF_001593425) genome as the reference. Conservation was quantified by calculating the length of the longest common subsequence (LCS) between each genome. Percent synteny was defined as the LCS length divided by the number of genes in the reference environment and expressed as a percentage.

### 2.4 RNA extraction, RT-PCR and RT-qPCR assays

An overnight culture of the *A. baumannii* WT strain grown in LB medium was diluted 1:100 in fresh medium and incubated at 37 °C until reaching the mid-exponential growth phase (OD_600_ ~ 0.4). Cells from 5 mL of each culture were harvested by centrifugation at 13,000 × *g* and resuspended in Tris-EDTA (TE) buffer, followed by treatment with lysozyme (50 mg/mL) for 10 min at 37 °C. Total RNA was extracted using the RNeasy Mini kit (Qiagen) according to the manufacturer’s instructions. Residual DNA contamination was removed by treatment with TURBO DNA-free™ (Invitrogen), and the absence of genomic DNA was verified by PCR.

Reverse transcription PCR (RT-PCR) assays were carried out using gene-specific oligonucleotides (listed in Supplementary Table S1) and the First-Strand cDNA Synthesis Kit (NZYtech). Gene expression was quantified by reverse transcription quantitative PCR (RT-qPCR) using the Lightcycler RNA Master SYBR Green I kit on a Lightcycler 480 instrument (LC480, Roche), according to the manufacturer’s instructions. RNA concentrations were normalized prior to analysis, and gene-specific primers were designed to amplify ~200 bp fragments of the target genes (listed in Supplementary Table S1). Relative gene expression levels were calculated using the 2^−ΔΔCT^ method (Schmittgen and Livak, 2008), with 16S rRNA as the housekeeping reference gene. Two independent biological replicates were performed for each experiment.

### 2.5 Protein expression, purification and electrophoretic mobility shift assay

The GntR transcriptional regulator gene (*AUO97_RS16340*) was amplified from the chromosomal DNA of *A. baumannii* ATCC 17978 WT by PCR using gene-specific primers (Supplementary Table S1) to generate an N-terminal 6 × His-tagged construct. The PCR products were purified, digested with the appropriate restriction enzymes, and ligated into the pUA1108 expression vector (Mayola et al., 2014). The resulting plasmid was introduced into *E. coli* DH5α cells by electroporation and correct in-frame fusion was verified by colony PCR and confirmed by sequencing [Macrogen (RRID:SCR_014454)].

For protein expression, the recombinant plasmid was subsequently introduced into *E. coli* BL21 (DE3) pLysS cells. The protein production was induced with 1 mM IPTG (Isopropyl *β*-D-1-thiogalactopyranoside, Nzytech), and cells were harvested by centrifugation and lysed by sonication. Cell lysates were clarified by centrifugation, and supernatants were incubated with BD TALON resin (Clontech) for affinity purification. His-tagged proteins were eluted with 10 mM imidazole, dialyzed, analyzed by SDS-PAGE, and quantified by the Bradford method.

The promoter region of interest was amplified by PCR from *A. baumannii* ATCC 17978 WT genomic DNA using specific primers (Supplementary Table S1). The resulting fragment was cloned into the pGEM-T vector (Promega), introduced into *E. coli* DH5α, and verified by colony PCR and sequencing (Macrogen). Digoxigenin (DIG)-labeled DNA probes were generated by PCR using DIG-labeled oligonucleotides (Supplementary Table S1).

For electrophoretic mobility shift assays (EMSAs) experiments, DNA-protein binding reactions contained 25 ng of DIG-labeled probe and either 0 or 10 μg of purified protein. Reaction mixtures were incubated for 30 min at 30 °C in EMSA buffer [20 mM Tris–HCl (pH 8), 50 mM KCl, 5% (v/v) glycerol, 1 μg bulk non-specific carrier DNA (salmon sperm DNA), 0.5 mM DTT, and 0.1 mg/mL BSA]. Protein-DNA complexes were resolved by non-denaturing polyacrylamide gel electrophoresis, transferred onto a Biodyne B nylon membrane (Pall Gelman Laboratory), and detected according to the manufacturer’s instructions (Roche) [Roche (Roche Cat# 11333062910, RRID:AB_2313639)]. When indicated, unlabeled competitor DNA was included in the reactions to assess binding specificity.

### 2.6 Knockout mutant construction and complementation

Gene inactivation in *A. baumannii* was performed as previously described (Aranda et al., 2010). An internal fragment of the target gene was amplified by PCR using gene-specific primers (Supplementary Table S1). The resulting product was cloned into the pCR-BluntII-TOPO vector (Invitrogen), which is non-replicative in *A. baumannii*, and propagated in *E. coli* DH5α. Recombinant plasmids (0.1 μg) were introduced into *A. baumannii* by electroporation, and transformants were selected on LB agar plates containing kanamycin. Successful gene disruption was confirmed by PCR and verified by sequencing (Macrogen). Mutant stability was assessed by serially passaging of cultures for ten consecutive generations in the absence of selective pressure, followed by plating on LB agar with or without kanamycin to confirm the maintenance of the mutation.

For mutant complementation, the target gene was PCR-amplified using primers containing *Xba*I restriction sites (Supplementary Table S1) and cloned into the pBAV1Gm-T5-gfp plasmid (Corral et al., 2021). The recombinant plasmid was first introduced into *E. coli* DH5α by electroporation. After verifying the correct construct by colony PCR and sequencing (Macrogen), the plasmid was transferred into the corresponding *A. baumannii* knockout mutant. To assess plasmid stability, complemented strains were plated on LB agar with and without gentamicin, confirming proper maintenance of the plasmid under selective and non-selective conditions.

### 2.7 Oxidative stress assay

The hydrogen peroxide survival assay was performed as previously described, with slight modifications (Aranda et al., 2011). Overnight cultures of the tested strains were diluted 1:50 into fresh Mueller-Hinton (MH) broth and exposed to H_2_O_2_ (final concentration, 30 mM) or an equivalent volume of phosphate-buffered saline (PBS) as a control. Cultures were incubated for 30 min at 37 °C with shaking, immediately serially diluted, and plated onto the appropriate agar plates. Survival was calculated as percentage of colony forming units (CFU) recovered from H_2_O_2_-treated cultures to the CFU obtained from the PBS-treated controls. All assays were performed in at least three independent experiments, each in triplicate.

### 2.8 Macrophage survival assay

The J774A.1 murine macrophage cell line was routinely maintained in Dulbecco’s Modified Eagle Medium (DMEM; PAN Biotech) (ATCC Cat# TIB-67, RRID:CVCL_0358), supplemented with 1% (v/v) fetal calf serum (FCS) (Merck), and 0.5% (v/v) penicillin–streptomycin (Merck). Cultures were incubated at 37 °C in a humidified atmosphere containing 5% CO_2_. Prior to each experiment, cells were examined under an inverted light microscope to verify confluency and ensure the absence of contamination or morphological abnormalities.

The macrophage survival assay was performed as previously described, with minor modifications (Fodah et al., 2014; Dumigan et al., 2022). Briefly, 5 × 10^5^ macrophages were seeded into 24-well plates and allowed to adhere overnight. Bacterial suspensions were prepared in DMEM and added to the wells at a multiplicity of infection (MOI) of 10–20 bacteria per macrophage. To synchronize infection, plates were centrifuged at 300 × *g* for 5 min at room temperature and then incubated for 1 h at 37 °C with 5% CO_2_. Following infection, the medium was replaced with DMEM containing 100 mg/L gentamycin to eliminate extracellular bacteria. After 1 h of incubation, wells were washed with PBS, and intracellular bacteria were released by treating cells with 0.1% Triton X-100 for 15 min. Recovered bacteria were serially diluted and plated on LB agar to quantify intracellular CFUs, which were compared to the initial inoculum to assess intracellular survival. All experiments were performed in triplicate, each with four technical replicates.

### 2.9 Antimicrobial susceptibility testing

Minimum Inhibitory Concentrations (MICs) of the tested antimicrobials and disinfectants were determined using the broth microdilution method in 96-well polystyrene microplates, following Clinical and Laboratory Standards Institute (CLSI) guidelines (Weinstein and Lewis, 2020). Standardized bacterial suspensions were prepared from fresh culture plates and adjusted to a 0.5 McFarland standard. Suspensions were then diluted 1:100 in MH broth (Merck), yielding a final inoculum of approximately 5 × 10^5^ CFU/mL per well after addition of the antimicrobial agents at the specified concentrations. Microplates were incubated at 37 °C for 24 h, and bacterial growth was assessed by measuring turbidity with a Multiskan™ FC Microplate Photometer (Thermo Fisher Scientific). Each MIC determination was performed in triplicate, across at least three independent biological replicates to ensure reproducibility.

### 2.10 Motility study

Surface-associated motility of the *A. baumannii* knockout mutants was assessed on modified LB agar plates (1% tryptone, 0.5% yeast extract, 0.5% NaCl, 0.5% glucose, and 0.5% Difco agar). Bacterial cultures were grown to the early stationary phase, and 5-μl aliquots were spotted at the center of freshly prepared plates (Pérez-Varela et al., 2019). Plates were incubated at 30 °C for 16–20 h, allowing the WT strain to reach the plate edge. All motility assays were performed in triplicate across at least three independent experiments. Representative images of the motility patterns were captured using a ChemiDoc™ XRS + imaging system (Bio-Rad).

### 2.11 Virulence experiments

The virulence of *A. baumannii* strains was assessed using the *G. mellonella* (wax worm larva) infection model, following a previously described protocol (Peleg et al., 2009). Ten larvae per experimental group were injected with 10 μL of a bacterial suspension in PBS (~10^5^ CFU) prepared from exponentially growing cultures. Inoculum concentrations were confirmed by colony counts on LB agar plates supplemented with appropriate antibiotics. A separate group of larvae injected with 10 μL of PBS served as a negative control. Larvae survival was monitored every 12 h over a 7-day period. All experiments were independently repeated at least three times.

### 2.12 Statistical analysis

Data were analyzed using two-tailed, one-way analysis of variance (ANOVA), followed by Tukey’s *post hoc* test for multiple-group comparisons. Survival curves from the *G. mellonella* killing assays were plotted using the Kaplan–Meier method, and differences in survival were evaluated using the log-rank test. Statistical significance was defined as *p* value of < 0.05 [GraphPad Prism (RRID:SCR_002798)].

## 3 Results

### 3.1 The GntR transcriptional regulator forms a polycistronic operon with the ABC efflux pump genes and functions as an activator

**Figure 1:**
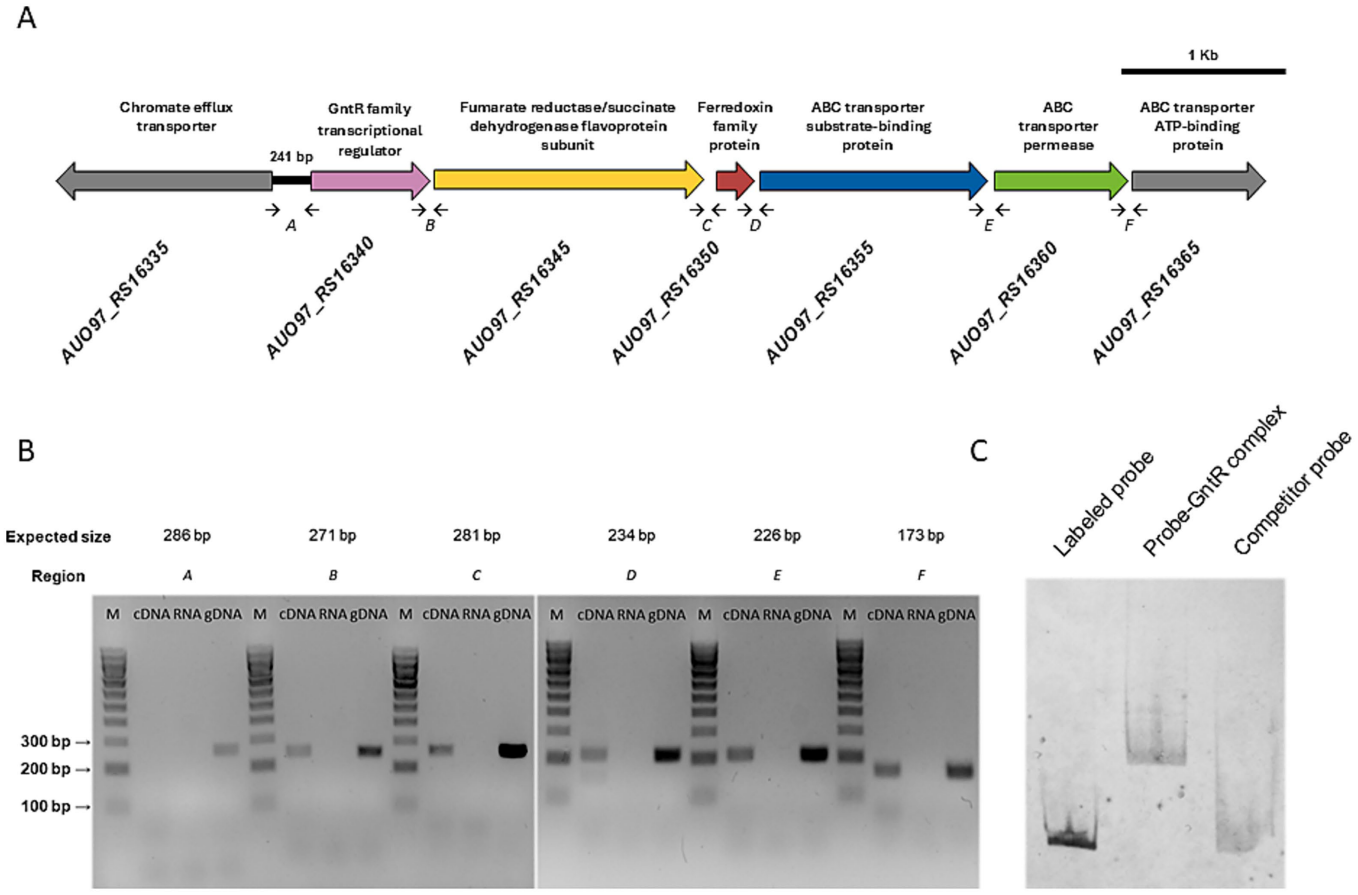
Characterization of the operon encoding the GntR-ABC efflux system in *A. baumannii* ATCC 17978. **(A)** Schematic representation of the GntR-ABC operon and surrounding gene cluster in the *A. baumannii* ATCC 17978 genome. Small arrows indicate the oligonucleotides used for RT-PCR analysis. The intergenic region between the *AUO97_RS16335* and *AUO97_RS16340* (241 bp) corresponds to the putative promoter region. Distances between adjacent protein-coding sequences within the operon are as follows: *RS16340-RS16345*, 28 bp; *RS16345-RS16350*, 74 bp; *RS16350-RS16355*, 19 bp; *RS16355-RS16360*, 34 bp; and *RS16360-RS16365*, 16 bp. **(B)** RT-PCR amplifications of the indicated intergenic regions showed (small black arrows). Each primer pair (Supplementary Table S1) was used in PCR reactions containing cDNA, RNA (negative control), or genomic DNA (positive control) from *A. baumannii* ATCC 17978 WT as templates. V Ladder (NzyTech) was used as the DNA size marker (M). **(C)** Representative EMSA of purified GntR protein and digoxigenin-labeled DNA probe containing the *AUO97_RS16335-RS16340* intergenic region. Competition assays were performed using 10-fold excess of unlabeled specific DNA as competitor.

In the *A. baumannii* ATCC 17978 strain, the GntR transcriptional regulator (encoded by *AUO97_RS16340*), together with several downstream genes, was initially identified among a set of genes overexpressed under oxidative stress conditions (Juttukonda et al., 2018). To further investigate the role of this transcriptional regulator, we analyzed the distribution and organization of *AUO97_RS16340* and its neighboring genes (Table 2). Regarding genomic organization, the transcriptional regulator is oriented opposite to *AUO97_RS16335*, which encodes a chromate efflux transporter, separated by 241 bp intergenic region (Figure 1A). Downstream of the GntR, five genes are arranged in the same transcriptional orientation: *AUO97_RS16345* and *AUO97_RS16350*, encoding a dehydrogenase subunit and a ferredoxin family protein, respectively, followed by an ABC efflux pump operon composed of *AUO97_RS16355* (substrate-binding protein), *AUO97_RS16360* (transporter permease) and *AUO97_RS16365* (ATP-binding protein) (Figure 1A).

To determine whether these genes form a single transcriptional unit, reverse transcription-PCR (RT-PCR) assays were performed. The results confirmed that the transcriptional regulator gene, together with the downstream ABC efflux pump subunit genes, is transcribed as a single polycistronic unit, independent of the adjacent chromate efflux transporter gene (Figure 1B). We also determine whether the GntR transcriptional regulator controls the downstream ABC efflux pump genes through promoter recognition and RT-qPCR. EMSAs were performed using the 241 bp intergenic region between *AUO97_RS16335* and the transcriptional regulator gene as a DIG-labeled DNA probe and the purified His-tagged GntR protein (Figure 1C). These experiments demonstrated that GntR specifically binds to the intergenic region upstream of the GntR-ABC efflux pump operon, confirming its role as a direct transcriptional regulator. Consistently, the expression level of *AUO97_RS16360* was reduced approximately two- to fourfold in RT-qPCR analyses in the GntR knockout mutant compared with the parental strain, indicating that GntR functions as a positive regulator of the operon.

### 3.2 The GntR-ABC efflux system is highly conserved and shows preserved genomic organization across *Acinetobacter baumannii* genomes

To evaluate the prevalence of homologs of the GntR regulator and associated ABC efflux pump genes in *Acinetobacter*, we analyzed the 1,479 complete genomes available on the NCBI, revealing that genes encoding them were present in 69.7–73.2% of genomes (Supplementary Table S2). However, their distribution was strongly species-dependent. Considering only species with more than 10 available genomes, the homologs were highly conserved in *A. baumannii* (*n* = 1,020), being present in 94.7–98.6% of genomes, and also prevalent in *A. nosocomialis* (84.6–96.2%). In contrast, these genes were rare or absent in other species, including *A. seifertii*, *A. indicus*, *A. haemolyticus*, *A. lwoffii* and *A. thermotolerans*. Intermediate or sporadic detection (3.7–16.9%) was observed in *A. pittii*, *A. johnsonii*, *A. junii* and *A. towneri*. Notably, *A. soli* (*n* = 13) lacked all of the genes except for *AUO97_RS16365*, which was present in 92.3% of the genomes (Supplementary Figure S1). On the other hand, the prevalence of these genes was lower across the 1,211 reference Gammaproteobacteria genomes (Supplementary Table S2). Interestingly, in the Enterobacteriaceae (*n* = 142), while ABC transporter homologs were detected in only 0–18.3% of strains, the GntR regulator was present in 63.4% of genomes, indicating substantial differences in the composition of its regulon.

Functional analysis of the *A. baumannii* ATCC 17978 GntR regulator and the associated ABC efflux pump proteins revealed distinct associated COG categories and Pfam domains. Specifically, the GntR regulator (AUO97_RS16340) was assigned to COG2188 and contained PF00392 and PF07702 domains. Among the ABC efflux pump proteins, AUO97_RS16355 belonged to COG0715 with PF13379, AUO97_RS16360 corresponded to COG0600 with PF00528, and AUO97_RS16365 was classified as COG1116 with PF00005.

In *A. baumannii*, the primary focus of this study, pairwise comparisons of all the identified homologous proteins revealed a high degree of conservation. Specifically, AUO97_RS16365 showed 99.1 ± 0.9% identity over 279 residues, AUO97_RS16340 had 99.4 ± 0.85% identity over 247 ± 1.2 residues, AUO97_RS16360 reached 98.4 ± 1.8% identity over 284 ± 1.2 residues, and AUO97_RS16355 displayed 98.8 ± 1.0% identity over 476 ± 0.9 residues. Similarly, the analysis (Supplementary Table S2) of the genomic context (5,000 bp flanking regions on each side of the *AUO97_RS16360* homologs) of *A. baumannii* encoding GntR and the corresponding ABC efflux pump proteins, revealed a remarkable conservation of gene content and synteny. For the majority of the strains examined (88.6%), all genes were present and the percent synteny value was 100%. However, a smaller subset of strains (11.4%) showed minor differences, with few genes absent or rearranged, leading to synteny values ranging from 63.6 to 90.9%, often corresponding to differences in similar COG identifiers (Supplementary Table S3). These findings highlight the strong conservation of this regulatory-efflux system among *A. baumannii* strains.

### 3.3 The GntR transcriptional regulator and its associated ABC efflux pump contribute to ROS detoxification *in vitro* and enhance survival within macrophages

Once the correlation of GntR and the ABC efflux pump was established, we next assessed their contribution to the oxidative stress response. To this end, the genes encoding the ABC permease (*AUO97_RS16360*, protein ID: WP_000096316.1), which confers functional specificity to the efflux pump by recognizing and exporting substrates, and the GntR regulator (*AUO97_RS16340*, protein ID: WP_000080816.1) were individually inactivated in *A. baumannii* ATCC 17978.

**Figure 2:**
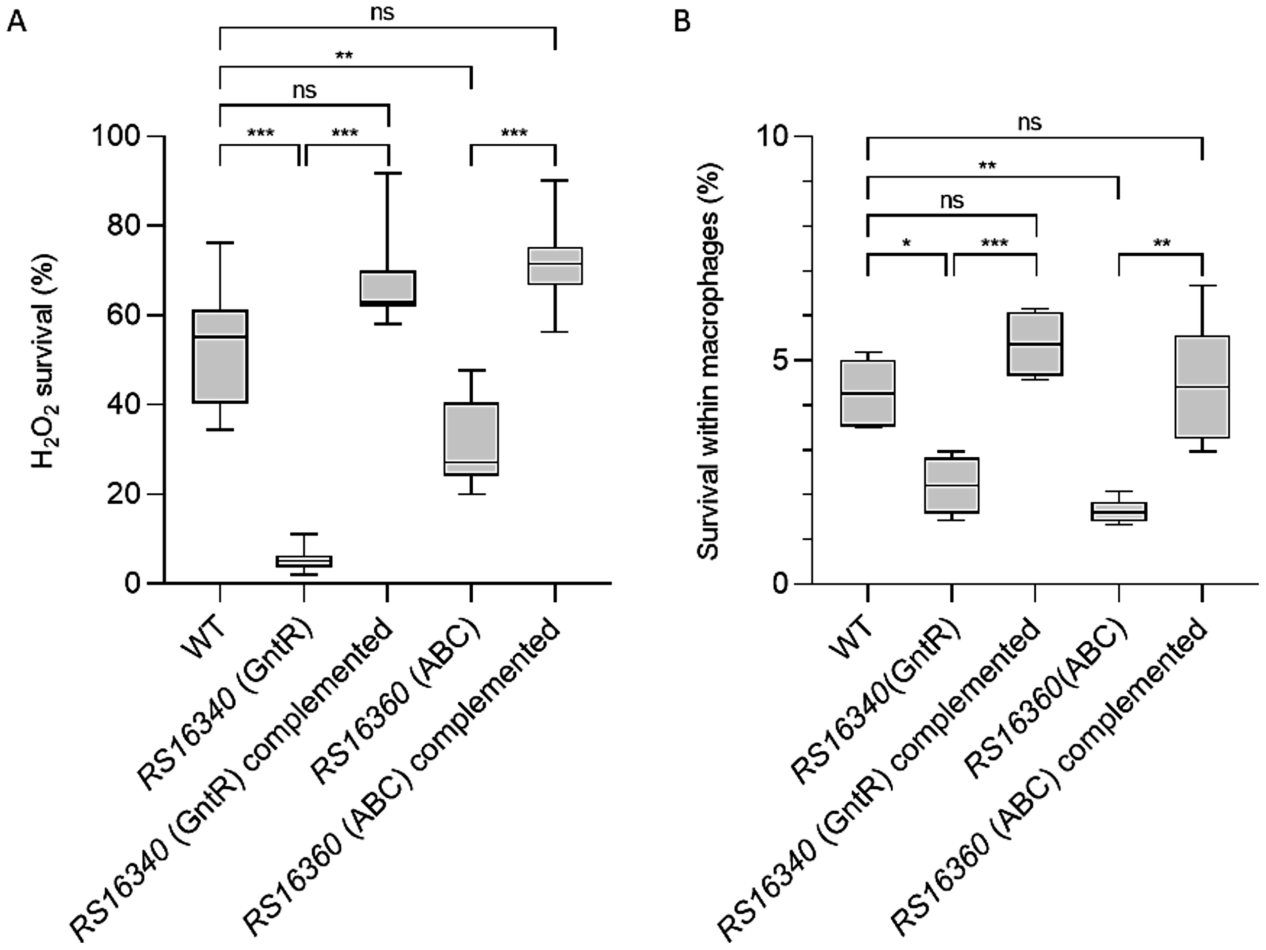
Impact of GntR regulator and ABC efflux pump on the *A. baumannii* survival under oxidative stress and within macrophages. **(A)** Survival of the *A. baumannii* ATCC 17978 WT, knockout mutants and complemented strains following oxidative stress exposure. Bacterial cultures at the stationary growth phase were treated with 30 mM H_2_O_2_ for 30 min. Relative survival was determined by viable cell counting. Error bars represent the standard deviation from three independent biological replicates; each performed in triplicate. **(B)** Relative intracellular survival of the indicated *A. baumannii* strains within J774A.1 murine macrophages. Values correspond to the average CFU recovered after macrophages infection, normalized to the initial bacterial inoculum. Error bars indicate the standard deviation from four technical replicates across three independent experiments. Asterisks denote statistical significance: *p* < 0.01 (*), *p* < 0.001 (**), and *p* < 0.0001 (***), (ns) indicate non-significant differences.

Oxidative stress survival assays that, after 30 min of treatment with H_2_O_2_, both GntR and ABC permease transporter knockout mutants display markedly increased sensitivity to exogenous H_2_O_2_, with survival rates below 10 and 30%, respectively (Figure 2A). These differences were statistically significant (*p* < 0.0001) compared with the *A. baumannii* WT strain, which exhibited approximately 60% under identical conditions. Furthermore, complementation of the mutants with the corresponding WT genes restored the survival rates to levels comparable to the parental strain (Figure 2A), confirming that the ABC efflux pump contributes to oxidative stress resistance and that the GntR regulator acts as a positive regulator of its expression.

H_2_O_2_ is a major ROS generated during the macrophage oxidative burst, representing one of the earliest and most potent macrophage-mediated bacterial killing (Cabiscol et al., 2000; Slauch, 2011; Winterbourn et al., 2016). Therefore, we further examined whether the GntR and the ABC permease knockout mutants were impaired in survival within macrophages. In infection assays using the J774A.1 murine macrophage cell line, both knockout strains exhibited significantly reduced intracellular viability compared with the WT strain (*p* < 0.01 and *p* < 0.001, respectively) (Figure 2B). Bacterial counts were approximately twofold lower for both mutants, while complementation restored intracellular survival to WT levels.

Together, these findings demonstrate that the GntR transcriptional regulator and its associated ABC efflux pump play are essential for *A. baumannii* to resist oxidative stress and to survive macrophage-mediated killing, underscoring their coordinated role in host-pathogen interactions.

### 3.4 The *Acinetobacter baumannii* GntR and ABC permease knockout mutants also display decreased antimicrobial resistance, impaired motility, and attenuated virulence

Given that efflux pumps are major contributors to antibiotic resistance in *A. baumannii* (Coyne et al., 2011; Verma et al., 2021), we investigated whether the identified ABC efflux pump and its corresponding regulator play a role in antimicrobial resistance. For this purpose, the MICs of multiple antimicrobial agents and disinfectants were determined for *A. baumannii* WT, knockout mutants, and their respective complemented strains. The tested compounds included representatives from diverse antibiotic classes, such as polymyxins, sulfonamides, *β*-lactams, cephalosporins, quinolones, aminoglycosides, and macrolides.

The results indicated that the GntR transcriptional regulator and the ABC efflux pump contribute modestly to broad-spectrum antimicrobial susceptibility. Among all compounds tested, only eight exhibited increased activity against one of the knockout mutants, whereas the remaining antimicrobials showed no differences compared with the WT strain. Specifically, the GntR knockout mutant exhibited a 2 or 4-fold reduction in growth in the presence of erythromycin, minocycline, tetracycline, amikacin, gentamicin streptomycin, apramycin and cefiderocol (Table 3). In contrast, the ABC permease mutant did not exhibit altered MICs for erythromycin and minocycline compared with the WT strain, suggesting that the GntR transcriptional regulator may control additional genes beyond the ABC efflux pump that contribute to antimicrobial susceptibility. Interestingly, most antibiotics with reduced MIC in both mutants belonged to the aminoglycoside family. Complementation restores MIC values to the WT levels in all cases, confirming the functional link between the GntR regulator and the ABC efflux system (Table 2).

**Table 3:** The Minimum Inhibitory Concentration (MIC, mg/L) of the tested antimicrobials for *A. baumannii* ATCC 17978 WT and the derivative knockout mutants.

Antimicrobial	ATCC 17978 (WT)	*RS16340* (GntR)	*RS16340* (GntR) Complemented	*RS16360* (ABC)	*RS16360* (ABC) Complemented
Minocycline	0.25	**0.125**	0.25	**0.25**	0.25
Tetracycline	4	**1**	4	**2**	4
Amikacin	8	**2**	8	**4**	8
Gentamicin	2	**0.5**	NA	**1**	NA
Streptomycin	64	**16**	64	**32**	64
Apramycin	16	**8**	16	**8**	16
Erythromycin	8	**4**	8	8	8
Cefiderocol	0.5	**0.25**	0.5	**0.25**	0.5
Colistin	1	1	1	1	1
Trimethoprim	16	16	16	16	16
Sulfamethoxazole	>256	>256	>256	>256	>256
Ampicillin	64	64	64	64	64
Ticarcillin	32	32	32	32	32
Ceftazidime	8	8	8	8	8
Cefotaxime	8	8	8	8	8
Ciprofloxacin	0.25	0.25	0.25	0.25	0.25
Levofloxacin	0.125	0.125	0.125	0.125	0.125
Novobiocin	16	16	16	16	16
Rifampicin	4	4	4	4	4
Chloramphenicol	64	64	64	64	64
DC	4,000	4,000	4,000	4,000	4,000
SDS	2,000	2,000	2,000	2,000	2,000
Acriflavine	16	16	16	16	16
Chlorhexidine	4	4	4	4	4

Differences compared to the WT strain are indicated in boldface.

NA, Not applicable; the pBAV1Gm-T5-gfp plasmid carries a gentamicin resistance cassette used for complementation.

**Figure 3:**
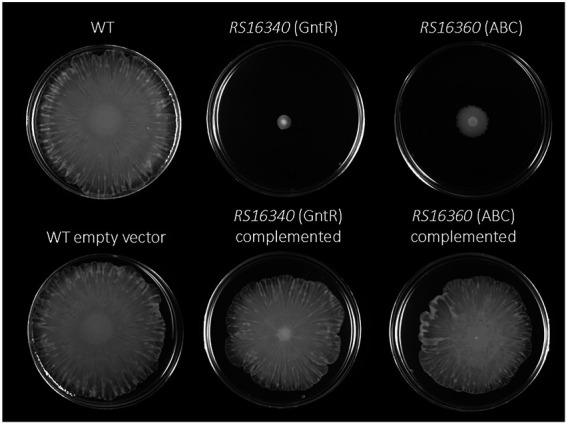
Surface-associated motility of the *A. baumannii* ATCC 17978 WT, GntR and ABC-efflux pump defective mutants. Representative images of motility on modified LB plates of the different strains are shown. WT carrying the empty vector, and complemented strains were also assessed as controls. Motility assays were performed in at least three independent experiments, all yielding reproducible results.

Beyond their role in antimicrobial resistance, efflux pumps have also been associated with multiple virulence-related processes (Knight et al., 2018). Therefore, we next assessed whether inactivation of the GntR regulator or the ABC efflux pump affected *A. baumannii* motility or virulence. Surface-associated motility assays revealed that the WT strain exhibited a robust spreading phenotype on modified LB agar plates (Figure 3). In contrast, the GntR mutant completely lost surface motility, and accordingly, the ABC permease mutant also showed impaired surface movement. Complementation of both mutants successfully restored surface-associated motility to levels comparable to the WT strain (Figure 3). It is worth noting that the introduction of the empty expression vector had undetectable effect on any strain, and growth curves analyses confirmed that all strains, including knockout and complemented derivatives, displayed similar growth kinetics in LB medium (Supplementary Figure S2), excluding growth rate differences as the cause of the motility defect.

**Figure 4:**
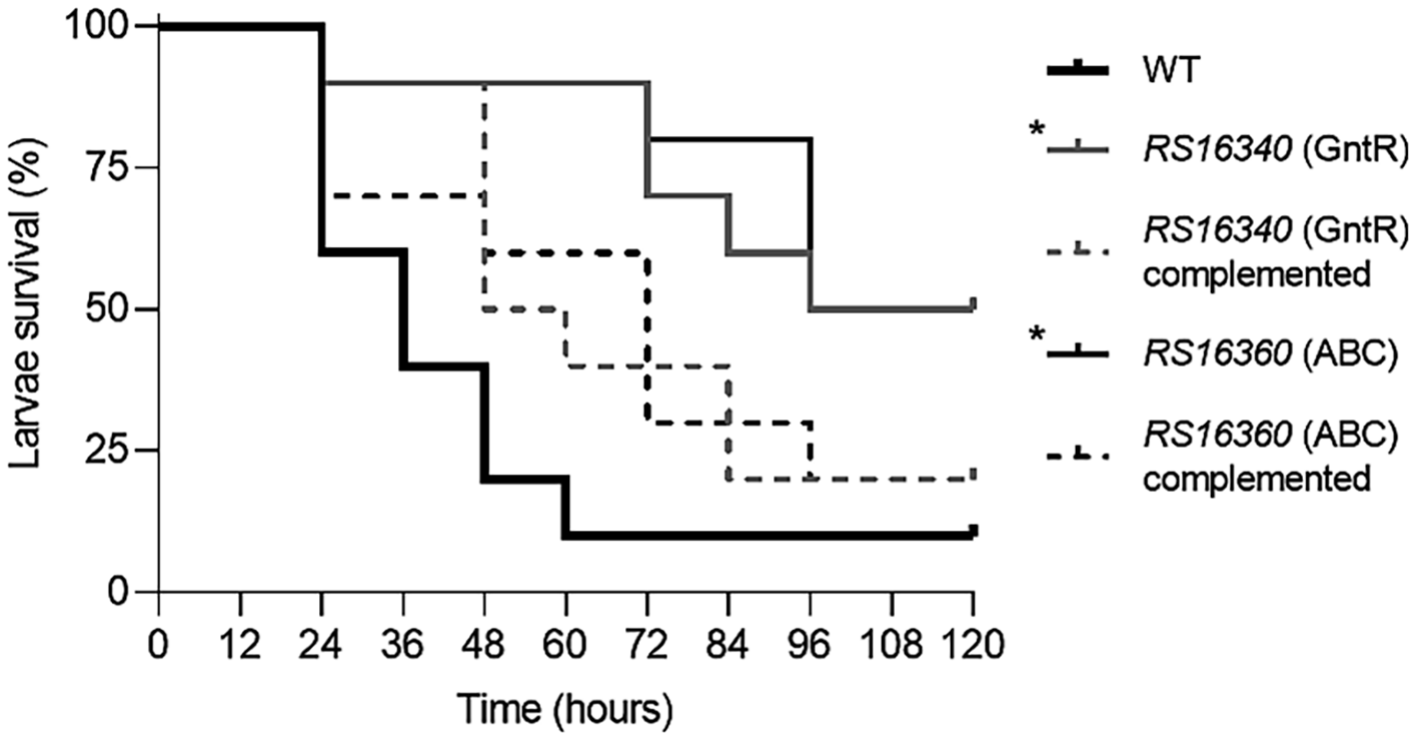
Virulence of the *A. baumannii* ATCC 17978 WT, knockout mutants and complemented strains. *G. mellonella* killing assays were performed using 10 larvae per group that were injected with 10 μL of a bacterial suspension, containing approximately 10^5^ CFU/mL of the indicated strain, or with PBS as a negative control (data not shown). The asterisk (*) denotes a statistically significant difference compared to the *A. baumannii* parental strain (*p* < 0.01). All *G. mellonella* infection experiments were performed in at least three independent biological replicates, yielding reproducible results. A representative Kaplan–Meier survival curve is shown.

Finally, the impact of efflux pump and its regulator inactivation on virulence was evaluated using the *G. mellonella* infection model (Peleg et al., 2009). The results revealed significant differences in larvae survival (*p* < 0.01) between the WT strain and both knockout mutants and the WT strain. Approximately 50% of the larvae inoculated with either the GntR or ABC permease mutant survived throughout the experiment, in contrast to the nearly complete mortality observed in the larvae inoculated with the WT strain (Figure 4). Moreover, complementation of the knockout mutants fully restored their pathogenic potential, as larvae inoculated with the complemented mutants exhibited survival rates comparable to those observed for the WT strain (Figure 4).

## 4 Discussion

*A. baumannii* is a highly adaptable opportunistic pathogen, well known for its persistence in healthcare environments and its capacity to withstand hostile conditions, antimicrobial treatments, and immune responses, while deploying multiple virulence factors that enhance its survival (Lee et al., 2017; Harding et al., 2018; Ibrahim et al., 2021). In this study, we characterized the role of the GntR family transcriptional regulator and ABC transporter permease, initially identified in transcriptional profiling of *A. baumannii* exposed to H_2_O_2_ as part of the canonical OxyR regulon (Juttukonda et al., 2018). Based on these data, it is assumed that these genes are likely associated with oxidative stress responses, supporting bacterial survival under fluctuating ROS levels both in environmental niches and within the host by contributing to a coordinated defense against H₂O₂-induced damage.

First, we demonstrated that the GntR transcriptional regulator is co-transcribed with the downstream ABC efflux pump genes, forming a single polycistronic transcriptional unit, and positively controls their expression through direct promoter binding. This genomic organization resembles that of other efflux systems in *A. baumannii*, where regulatory genes frequently located close to the efflux pump operons they control, either transcribed in opposite direction or, as in this case, within the same operon, and can function as transcriptional activators or repressors (Liu et al., 2018; Leus et al., 2020; Gaona et al., 2024; Wimalasekara et al., 2025). Remarkably, the genes encoding the GntR regulator and the associated ABC efflux pump are highly conserved across *Acinetobacter*, being detected in 69.7–73.2% of the 1,479 complete genomes analyzed, but showing a strong species-dependent distribution. In particular, they are nearly ubiquitous in *A. baumannii* (94.7–98.6% of 1,020 genomes), where they display very high sequence identity (>98%) and conserved genomic organization, whereas they are sporadic or absent in most other *Acinetobacter* species and considerably less prevalent across reference Gammaproteobacteria, including *Enterobacteriaceae*. These data suggest a lineage-associated role and highlight their potential importance for bacterial fitness and adaptation in *A. baumannii*. Likewise, several other efflux pump genes, including *adeB*, *adeF*, *adeG, adeJ*, *macB*, *abeS* and *sxtP* are frequently detected in clinical isolates (Liu et al., 2018; Kaviani et al., 2020; Gaona et al., 2024).

Through gain- and loss-of-function experiments, we demonstrated that the GntR transcriptional regulator and its associated ABC efflux pump are involved not only in the oxidative stress response but also in several pathogenesis-related processes, including antimicrobial susceptibility, motility, and virulence. The GntR family of transcriptional regulators is one of the most abundant regulatory families in bacteria (Suvorova et al., 2015; Liu et al., 2021). Although initially characterized in *Bacillus subtilis* as repressors of the gluconate operon, and frequently described as transcriptional repressors (Liu et al., 2021), subsequent studies indicates that several GntR members function as transcriptional activators depending on their regulatory context. Activator roles have been reported in diverse physiological processes, including metabolic regulation, such as TauR-mediated activation of taurine utilization genes in *Rhodobacter capsulatus* (Wiethaus et al., 2008), and CitO-dependent activation of citrate metabolism genes in *Enterococcus faecalis* (Blancato et al., 2008). In addition, GntR regulators have been shown to positively regulate antibiotic resistance determinants in *Mycobacterium* spp., where a GntR-type transcription factor enhances isoniazid resistance by controlling permease expression (Hu et al., 2015), as well as virulence and stress-response pathways in *X. campestris* (Su et al., 2016). For instance, HpaR1 from *X. campestris* activates genes involved in hypersensitive response, polysaccharide biosynthesis, extracellular enzyme production, motility, and oxidative stress tolerance (Su et al., 2016). Together, these examples demonstrate that activator functions are well established within the GntR family. ABC efflux pumps typically comprise three components: an ATPase, a permease and the substrate binding protein. While all components are required for full transport activity, the permease plays a central role in substrate translocation across the membrane (ter Beek et al., 2014). Consequently, inactivation of the transmembrane permease alone is often sufficient to disrupt efflux pump function, enabling the analysis of its contribution to physiological and virulence-associated processes, including oxidative stress (Crow et al., 2017; Pérez-Varela et al., 2019; Kaviani et al., 2020; Shirshikova et al., 2021).

Oxidative stress represents a major challenge for pathogenic bacteria exposed to ROS generated by host immune cells, particularly during macrophage infection (Daugherty et al., 2012; Sato et al., 2019; Cesinger et al., 2021; Scribano et al., 2025). To counteract ROS toxicity, bacteria rely on an array of detoxification enzymes, such as superoxide dismutases (Staerck et al., 2017), catalases (Flores-Cruz and Allen, 2011), and peroxidases (Jittawuttipoka et al., 2009; Sun et al., 2016), whose expression is often coordinated by global regulators including OxyR, SoxR and PerR (Hishinuma et al., 2006; Seib et al., 2007; Imlay, 2015; Juttukonda et al., 2018).

Our findings reveal that the polycistronic transcriptional unit, which includes six genes forming a regulatory-efflux system, is part of this broader ROS-defense network in ATCC17978 strain. Similar mechanisms have been reported in other pathogens, where oxidative stress triggers efflux pump overexpression as a protective strategy against ROS (Fernando and Kumar, 2013). For instance, the MacAB efflux pump in *Serratia marcescens* enhances survival under oxidative stress and confers resistance to aminoglycosides and polymyxins and plays a key role in bacterial survival under oxidative stress (Shirshikova et al., 2021). Interestingly, susceptibility to cefiderocol, a recently introduced siderophore-conjugated cephalosporin with promising therapeutic potential (Raro et al., 2026), was modestly increased in the *A. baumannii*-derived mutants. In *Mycobacterium tuberculosis*, the major facilitator superfamily (MFS) efflux pump Rv1258c contributes to both drug resistance and oxidative stress response and iron metabolism through the ESX-3 secretion system (Sun et al., 2024). Similarly, in *Campylobacter jejuni*, the CosR transcriptional regulator governs oxidative stress by modulating genes such as KatA, and promotes the extrusion toxic compounds through expression control of the CmeABC efflux pump (Lin et al., 2002). Collectively, these examples highlight the dual role of efflux systems in *A. baumannii* ATCC 17978, not only in antibiotic susceptibility but also in maintaining redox homeostasis, emphasizing their evolutionary importance in bacterial survival under oxidative stress.

The emergence of AMR in *A. baumannii* is multifactorial, involving enzymatic drug inactivation, target site modification, reduced cell permeability, and active efflux extrusion (De Oliveira et al., 2020). Among these mechanisms, efflux pumps play a central role in conferring multidrug resistance (Coyne et al., 2011; Knight et al., 2018). In the present study, our results indicate that the ABC efflux pump regulated by the GntR transcriptional regulator contributes to drug tolerance. Previous works have demonstrated that *A. baumannii* encodes multiple efflux systems implicated in multidrug resistance. For instance, the AdeABC resistance nodulation division (RND) efflux pump confers resistance to aminoglycosides, fluoroquinolones, tetracyclines, chloramphenicol, and macrolides (Magnet et al., 2001). Similarly, the SxtP MFS pump, together with its transcriptional activator SxtR mediates resistance to sulfamethoxazole/trimethoprim, while also modulating bacterial motility and virulence (Gaona et al., 2024). Notably, even in cases where aminoglycoside resistance is not driven by modifying enzymes (Poole, 2005), efflux pumps have been shown to be the predominant mechanism. This has been documented in clinical isolates of *E. coli*, *Proteus* spp., *Providencia stuartii*, and *S. marcescens* (Ramirez and Tolmasky, 2010; Shirshikova et al., 2021).

Bacterial motility and pathogenesis are often associated with efflux pump activity (Knight et al., 2018). In this study, both the ABC efflux pump and the GntR regulator significantly affected surface-associated motility and virulence in the *G. mellonella* invertebrate model. Surface-associated motility in *A. baumannii* has been linked to the production and release of extracellular polymeric molecules and signaling compounds, which can be modulated by efflux pump activity (Jeong et al., 2024). These efflux systems may contribute to motility by exporting surfactant-like or signaling molecules required for surface movement and, hypothetically, the observed phenotype in the *A. baumannii* knockout mutants could be associated with impaired extrusion of such molecules. These observations are consistent with previous studies suggesting that efflux systems contribute to motility and virulence-related traits across diverse bacterial species. In *A. baumannii*, efflux transporters from at least five superfamilies, including ABC pumps, have been associated with the regulation of surface-associated motility and virulence (Pérez-Varela et al., 2019; Gaona et al., 2025; Xie et al., 2025). In other pathogens, AMR-mediated efflux pumps are frequently intertwined with motility and virulence. For instance, the AcrAB-TolC pump in *E. coli* contributes not only to multidrug resistance but also to host colonization (Fernando and Kumar, 2013). Similarly, the previous described MacAB efflux pump in *S. marcescens* modulates bacterial motility and oxidative stress (Shirshikova et al., 2021). In *P. aeruginosa*, inactivation of the MuxABC-OpmB system leads to impaired motility and reduced virulence (Yang et al., 2011). Comparable phenotypes have also been described in *Klebsiella pneumoniae* and other multidrug-resistant pathogens, where efflux activity facilitates immune evasion (Knight et al., 2018; Yazgan et al., 2018). Collectively, these findings highlight that efflux systems often coordinate drug resistance, motility, and virulence, thereby integrating environmental sensing with pathogenicity.

In conclusion, this study identifies a GntR family transcriptional regulator and its associated ABC efflux pump as components of a regulator/efflux system in *A. baumannii* ATCC 17978 that connects oxidative stress adaptation, antimicrobial susceptibility, and virulence-associated traits. We demonstrate that the GntR transcriptional regulator, together with its associated ABC efflux pump, contributes to ROS tolerance, surface-associated motility, and pathogenicity in the *G. mellonella* infection model. Although *A. baumannii* exhibits considerable genomic plasticity and our experimental work was restricted to the ATCC 17978 background, bioinformatic analyses indicate that these components are highly conserved across available *A. baumannii* genomes. Therefore, while further validation in clinically representative high-risk lineages is warranted, these findings suggest that this regulatory/efflux system may represent a promising target for therapeutic strategies aimed at disabling bacterial defense mechanisms and attenuating virulence.

## Data Availability

The original contributions presented in the study are included in the article/Supplementary material, further inquiries can be directed to the corresponding authors.

## Author’s note

The authors would like to acknowledge the official titles of the mentioned contributors as follows: Jordi Barbé is Emeritus Professor; Susana Campoy and Jesús Aranda are Serra Húnter Fellows.
