# Supplementary material for: A novel GntR-ABC efflux system mediates oxidative stress response, drug resistance, motility and virulence in Acinetobacter baumannii ATCC 17978
Source: Front Microbiol. 2026 Apr 9;17:1748186. doi: 10.3389/fmicb.2026.1748186 (PMC13104710; doi:10.3389/fmicb.2026.1748186)
Supplement: Supplementary file 1 [file Table_1.docx]

Supplementary Material

# Supplementary Figures and Tables

## Supplementary Figure 1.


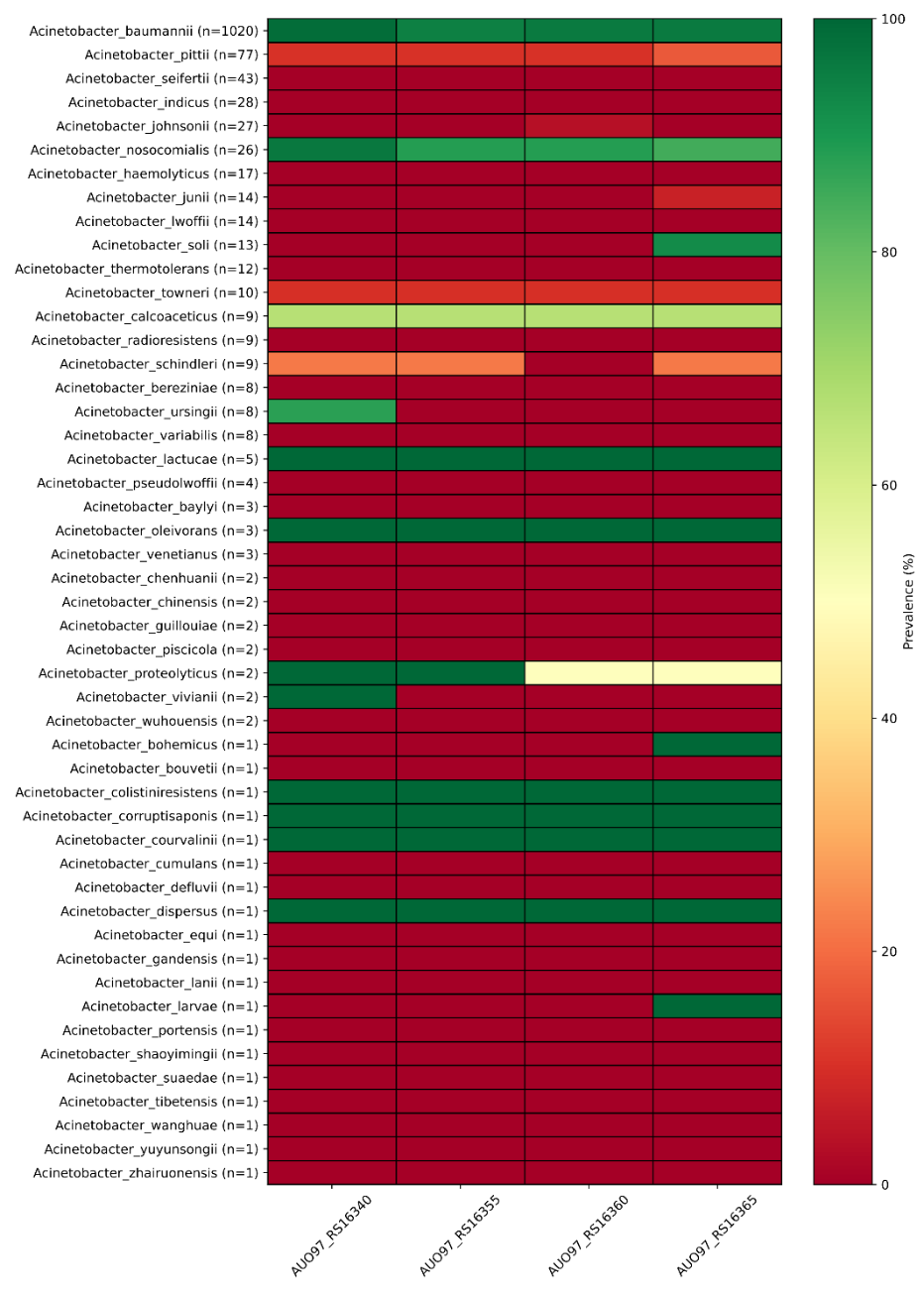


**Supplementary Figure 2.** Heatmap depicting the prevalence of homologs of AUO97_RS16340 (GntR) and the associated ABC efflux system proteins (AUO97_RS16355, AUO97_RS16360, and AUO97_RS16365) across all complete Acinetobacter genome assemblies analyzed. The number of genomes examined per species is indicated next to each species’ name. Color ranges from red (0 % prevalence) to green (100 % prevalence).

## Supplementary Figure 2.

**Supplementary Figure 2.** Growth curves of the WT and knockout mutant strains monitored over 24 hours by measuring optical density (OD_600_) every hour. Error bars indicate the standard deviation of the mean from three replicates per strain.

## Supplementary Table 1. Oligonucleotides used in this work.

| Name | Sequence (5’ → 3’) | Application |
| --- | --- | --- |
| RS16335-RS16340_Fw | GGGGTATTTAATTGTTTTTGTTGC | RT-PCR assays |
| RS16335-RS16340_Rv | CTTGGGGTAGCTGGCTCAC | RT-PCR assays |
| RS16340-RS16345_Fw | CTCCGGTATTACGCGTTG | RT-PCR assays |
| RS16340-RS16345_Rv | CATTCGCTTTTTCAATGAGC | RT-PCR assays |
| RS16345-RS16350_Fw | CGGATTGGTTCTGTCACGCG | RT-PCR assays |
| RS16345-RS16350_Rv | CATCTACACAGACAGTGCATCC | RT-PCR assays |
| RS16350-RS16355_Fw | GTTGGTATTGCATGCCTTGTG | RT-PCR assays |
| RS16350-RS16355_Rv | GTTAATGGTCGTGTCTTGTG | RT-PCR assays |
| RS16355-RS16360_Fw | GACTTTAAAGCTATTCAGCTTG | RT-PCR assays |
| RS16355-RS16360_Rv | CTGAGCAAGTCCTTGCCAAATG | RT-PCR assays |
| RS16360-RS16365_Fw | GGTATGTTTAGCAGTACATTGG | RT-PCR assays |
| RS16360-RS16365_Rv | CGAAATTAACCCGATCTAATGCCTG | RT-PCR assays |
| pUA1108-RS16340_Fw | actgCATATGAGCCAGCTACCCCAAGATCCC | Cloning for protein overexpression |
| pUA1108-RS16340-His_Rv | actgGGATCCTTA**ATGATGATGATGATGATG**gccgccgccACGATGAATACGTAAACGGTAC | Cloning for protein overexpression |
| -108pUA1108 | CCGACATCATAACGGTTC | Sequencing primer for pUA1108 vector |
| +128pUA1108 | AGACAAGCTGTGACCGTC | Sequencing primer for pUA1108 vector |
| pRS16340_Fw | ATTATCCCCTAAAAAATAATATTAA | Cloning for promoter amplification |
| pRS16340_Rv | AACCTGTTATAACAACTTGA | Cloning for promoter amplification |
| M13FpUC* | GTTTTCCCAGTCACGAC | Sequencing primer for pGEMT and pCR-BluntII-TOPO vectors |
| M13RpUC* | CAGGAAACAGCTATGAC | Sequencing primer for pGEMT and pCR-BluntII-TOPO vectors |
| Mut-RS16340_Fw | cagtGAATTCGTAAATGGTGAATATGCCG | Mutant construction |
| Mut-RS16340_Rv | cagtGAATTCCATACACATTGGCTTTAAC | Mutant construction |
| Com-RS16340_Fw | cagtTCTAGAGTGAGCCAGCTACCCCAAG | Mutant complementation and verification |
| Com-RS16340_Rv | cagtTCTAGATTAACGATGAATACGTAAACGG | Mutant complementation and verification |
| Mut-RS16360_Fw | cagtGAATTCGTAGTTTAGCCCAGACAAAACC | Mutant construction |
| Mut-RS16360_Rv | cagtGAATTCCAGAATCGCTAAAGGAATCC | Mutant construction |
| Com-RS16360_Fw | actgTCTAGAATGAGTAGTTTAGCCCAGAC | Mutant complementation and verification |
| Com-RS16360_Rv | actgTCTAGATTGATACCATGGAGTAAATC | Mutant complementation and verification |
| pBAV_Fw | CACTGTTCCTTGCATTCTA | Sequencing primer for pBAV1Gm-T5-gfp |
| pBAV_Rv | ATTGGGACAACTCCAGTGAA | Sequencing primer for pBAV1Gm-T5-gfp |
| 16S_Fw | GATGCAACGCGAAGAACC | RT-qPCR assays |
| 16S_Rv | CGTAAGGGCCATGATGACTT | RT-qPCR assays |
| RS16360-RT-qPCR_Fw | GTTATTCATGCGCTTTGGGC | RT-qPCR assays |
| RS16360-RT-qPCR_Rv | CTGCTGGGATTGGACGTAAT | RT-qPCR assays |

Sequences recognized by restriction endonuclease sites are underlined (CATATG: *Nde*I, and GGATCC: *BamH*I GAATTC: *EcoR*I, and TCTAGA: *Xba*I), 6×His tags are indicated in bold, and Gly spacers and restriction site tails in lower case.

*When needed, primers used were labeled with DIG at 5′ for EMSAs.

## Supplementary Table 2. Summary of *in silico* genomic analysis of *AUO97_RS16340* (GntR Transcriptional Regulator) and *AUO97_RS16355*, *AUO97_RS16360* and *AUO97_RS16365* (ABC Efflux Pump) genes in representative *Acinetobacter* species (n = number of analyzed genomes) (n = 1,479) compiled from NCBI database.

|  | Prevalence (%) | | | | |
| --- | --- | --- | --- | --- | --- |
| n = 1,479 genomes | | *AUO97_RS16340*  (*A1S_1717*) | *AUO97_RS16355*  (*A1S_1720*) | *AUO97_RS16360*  (*A1S_1721*) | *AUO97_RS16365*  (*A1S_1722*) |
| *Acinetobacter* spp. | | 73.16 | 69.71 | 70.52 | 72.14 |
|  | | **Prevalence** (%) | | | |
| n = 1,020 genomes | | *AUO97_RS16340*  (*A1S_1717*) | *AUO97_RS16355*  (*A1S_1720*) | *AUO97_RS16360*  (*A1S_1721*) | *AUO97_RS16365*  (*A1S_1722*) |
| *Acinetobacter baumannii* | | 98.63 | 94.71 | 95.98 | 96.08 |

The raw data of the corresponding prevalence percentages are provided in the Table S2 spreadsheet.

## Supplementary Table 3. Genomic contexts of the *Acinetobacter baumannii* ABC efflux system homologs, including protein identifier, functional annotation, COG assignment and sequence length. Gene content conservation and percent synteny relative to *A. baumannii* ATCC 17978 are also reported.

The raw data of the corresponding genomic contexts are provided in the Table S3 spreadsheet.
